# Analysis of intraocular positions of posterior implantable collamer lens by full-scale ultrasound biomicroscopy

**DOI:** 10.1186/s12886-018-0783-5

**Published:** 2018-05-09

**Authors:** Xi Zhang, Xun Chen, Xiaoying Wang, Fei Yuan, Xingtao Zhou

**Affiliations:** 1 Department of Ophthalmology, Zhongshan Hospital of Fudan University, Shanghai, People’s Republic of China; 2 Department of Ophthalmology, Eye and ENT Hospital of Fudan University, Shanghai, People’s Republic of China; 3 Department of Ophthalmology, Myopia Key Laboratory of the Health Ministry, Shanghai, People’s Republic of China

**Keywords:** High myopia, Phakic intraocular Lens, Implantable Collamer Lens, Ultrasound biomicroscopy, Vault

## Abstract

**Background:**

To analyze the positions of intraocular posterior Implantable Collamer Lens (ICL) and its possible relationship with vault.

**Methods:**

This cross-sectional study included 72 patients with high myopia (134 eyes) who were followed up after phakic intraocular lens implantation. The postoperative time ranged from 1 week to 7 years. We obtained the images of ICL by using Compact Touch STS UBM and observed the position of ICL in posterior chamber and ciliary sulcus. The horizontal lines vault was measured and recorded.

**Results:**

There were various positions in the posterior chamber as observed by full-scale ultrasound biomicroscopy and the haptics were inserted at different positions. -Eight seven eyes (64.9%) that obtained ideal vault, 29 eyes (21.6%) had insufficient vaults and 18 eyes (13.4%) had excessive vault. The vault with various positions of haptics was in ideal range (250 μm–750 μm) almost in each group. Three eyes in this study with haptics on the top of ciliary sulcus obtained excessive vault (mean vault, 850.00 ± 70.71 μm) and one eye appeared one side haptics pushing forward the iris. Among five eyes (3.7%) with iridociliary body cysts, three eyes (60%) obtained ideal vault. One eye (0.7%) with ICL decentralization after implantation surgery had an ideal vault, but the patient had serious glare.

**Conclusions:**

Though ICL in the posterior chamber had different positions and the haptics in most cases were not in the ciliary sulcus, the postoperative vault was almost in the ideal range.

## Background

Phakic intraocular lens (PIOL) implantation is a technique to correct high ametropia as the condition cannot be corrected by laser in situ keratomileusis (LASIK). The implantable collamer lens (Visian ICL; STAAR Surgical, Nidau, Switzerland) is a posterior chamber phakic IOL and was approved by FDA in December 2005 for commercial use in the United States for myopia -3D to -20D [1]. It is made of a porcine collagen/hema copolymer material which is thin, flexible, and hydrophilic. It is 7.0 mm wide and the length of myopia model varies from 11.5 to 13.0 mm. The diameter of the optic zone varies from 4.5 to 5.5 mm according to the diopter power of the lens. The Toric implantable collamer lens (TICL) correct − 3.00 to − 6.00D astigmatism [2]. ICL/TICL is most widely adopted for correction of high myopia in the world due to their safe and effective strategies than laser corneal surgery [3–7].

Ultrasound biomicroscopy is a high-frequency ultrasound technology that provides information of posterior iris, which cannot be evaluated through slit-lamp microscopy or Pentacam anterior segment analyzer before and after ICL/TICL implantation. Preoperatively, ultrasound biomicroscopy is helpful in discovering the existence of post-iris anomalies [8–10]. Postoperatively, ultrasound biomicroscopy evaluates the appropriate position of phakic IOL of posterior chamber, including the optical zone, bilateral haptics and relation to iris, crystalline lens, zonules and ciliary body [11, 12].

As we know, the ideal phakic IOL position show the optical zone located on the center of the pupil, and both haptics on the sulcus with an appropriate vault (250 μm -750 μm). Because excessive (> 750 μm) or insufficient (< 250 μm) vault lead to complications such as pigmentary loss, glaucoma or cataract, and the stability of vault becomes the major concern after ICL implantation [13]. The ICL dislocation usually results in an inappropriate vault [14]. To our knowledge, the relationship between different kinds of dislocation of ICL haptics and vault has not been reported till date. The purpose of this study is to evaluate the various positions of ICL haptics after implantation in high myopic eyes by capturing the images using full-scan ultrasound biomicroscopy and its effect on the vault.

## Methods

### Subjects

This cross-sectional study included 72 patients with high myopia (134 eyes) who were followed up after phakic intraocular lens implantation in the Eye and ENT Hospital of Fudan University from May to July in 2014. The mean age was 29.78 ± 7.61 years old (range 19–48 years old). The preoperative spherical diopter ranged from − 22.50D to − 5.50D and the mean spherical equivalent was − 13.32 ± 5.10D. The postoperative time ranged from 1 week to 7 years. Before surgery, all patients had a complete ophthalmic examination, such as uncorrected visual acuity (UCVA), best corrected visual acuity (BCVA), manifest and cycloplegic refraction, anterior chamber depth (ACD), corneal keratometry, intraocular pressure, retinal examination through a dilated pupil and ultrasound biomicroscopic examination. The inclusion criteria were as follows: spectacles and/or contact lens intolerance, stable refraction for at least 1 year before preoperative examination, the endothelial cell density (ECD) ≥2100 cell/mm, anterior chamber depth ≥ 2.8 mm, no history of cataract, glaucoma, uveitis, uncontrolled diabetes, collagen vascular disease, or previous intraocular surgery.

### ICL size calculation

Size of the myopic lens was determined by the horizontal white-to-white (WTW) and the anterior chamber depth (ACD) measurements. The lens size was calculated by adding 0.5 mm to the white-to-white measurement when eyes exhibited an ACD between 2.8 and 3.5 mm and 1.0 mm to the white-to-white measurement when the ACD was greater than 3.5 mm. All lens power calculations were performed by STAAR Surgical Company.

### Surgical procedure

At least 1 week before ICL implantation, patients received two peripheral laser Nd:YAG iridotomies at 10:30 and 1:30 clock positions. On the day of surgery, 2.5% phenylephrine for mydriasis and 0.4% oxybuprocain hydrochloride eye drops were applied to the operative eye before surgery. All ICL/TICL implantations into the posterior chamber were performed with an injector cartridge designed by STAAR Surgical through a 3.2 mm corneal tunnel incision in the horizontal meridian using peribulbar anesthesia. The anterior chamber was filled with sodium hyaluronate 1% (Provisc), which was completely removed at the end of surgery. During TICL implantation, the surgeon marked the zero horizontal axis at the 3- and 9-o’clock limbus using a marking pen with the patient sitting upright at a slit lamp. The surgeon also used a Mendez ring to measure the required rotation from horizontal during the surgical procedure and the lens was rotated to the required axis using a modified intraocular spatula. Tobramycin eye drops were used four times a day for 7 days, 0.1% fluorometholone eye drops were instilled six times a day from the second day after surgery and minus one time every three days for one month.

### Postoperative full-scale ultrasound biomicroscopy examination

**Fig. 1:**
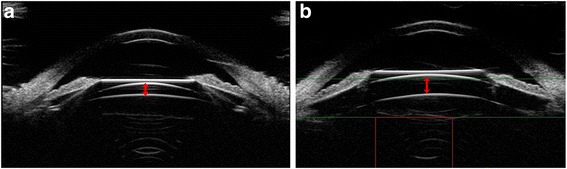
Images of normal anterior segment on horizontal (**a**) and vertical (**b**) meridians as measured by Compact Touch STS UBM. Arrows showed the vault of horizontal and vertical meridian, respectively

**Fig. 2:**
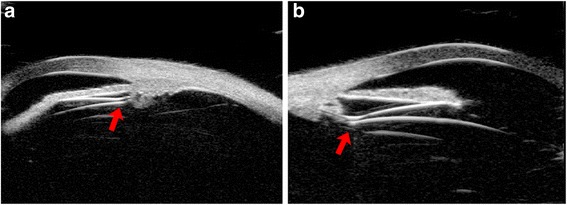
Images of ICL haptics position on temporal (**a**) and nasal (**b**) as measured by Compact Touch STS UBM. Arrows showed the haptics position

During the follow-up period, all subjects underwent postoperative Compact Touch STS UBM (Quantel Medical, France) examination whose linear scanning frequency is 50 MHz; scanning depth and width is 9 *16 mm; the resolution of axial and vertical is 35 μm, 60 μm, respectively. Its reliability has been reported in the previous study [15]. Examinations were performed using an eyecup filled with methylcellulose solution after topical oxybuprocaine hydrochloride was instilled to anesthetize the cornea, while the subjects were asked to fixate on a ceiling target and keep head still in the spine position. The examiner adjusts the probe perpendicular to the eyes, choose the UBM/STS pattern and acquires vertical (6 o’clock − 12 o’clock) and horizontal (3 o’clock - 9 o’clock) lines images. The patients were then asked to gaze at the nasal and temporal angles for acquiring images of ICL haptics in ciliary sulcus, and record the horizontal vault. Figs 1 and 2 showed the images of normal anterior segment taken by UBM after phakic IOL implantation as measured by Compact Touch STS UBM. All image acquisitions were completed by the same physician.

## Results

**Fig. 3:**
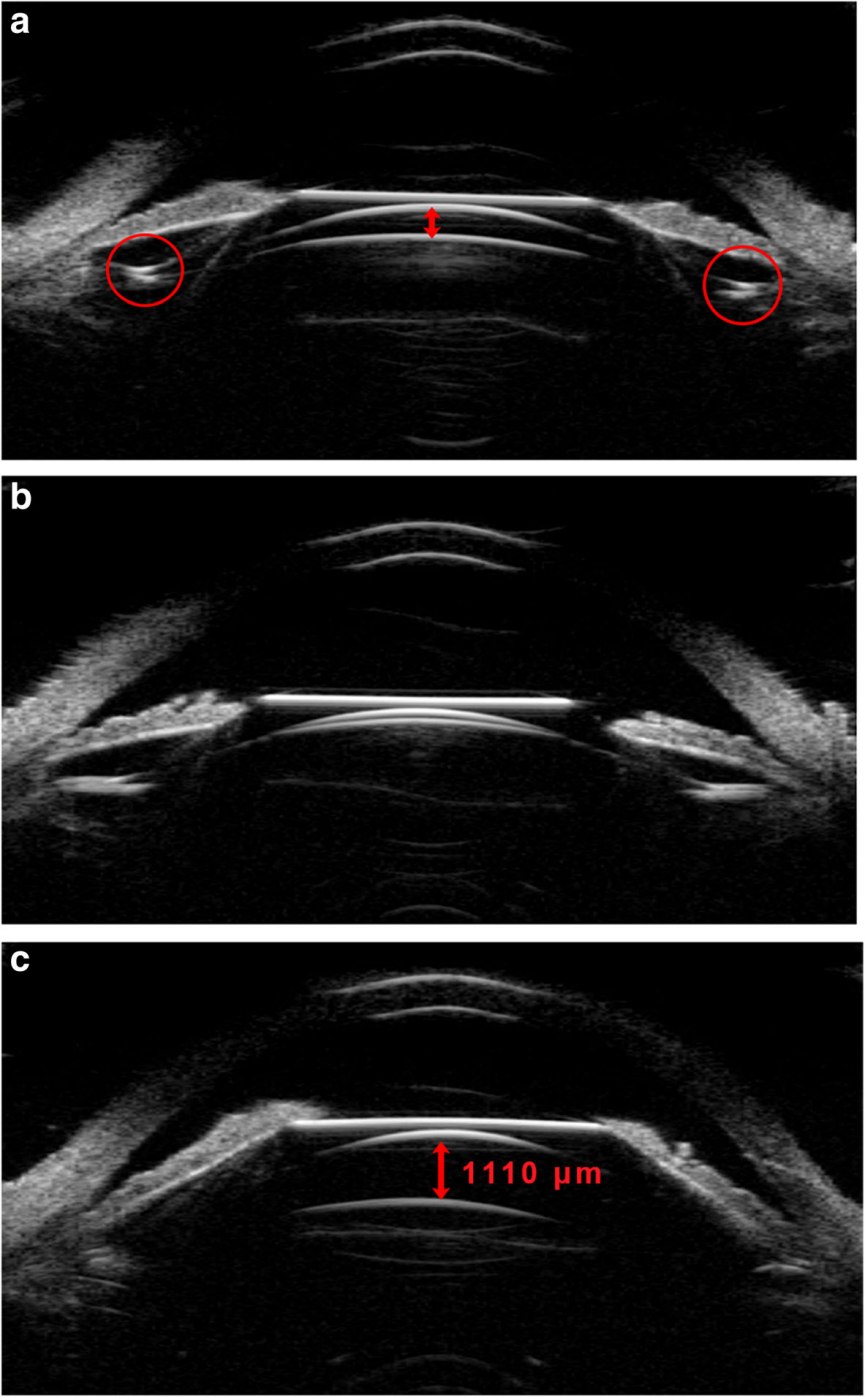
UBM images of ideal ICL posterior chamber position after implantation. **a** UBM images of one patient with ideal vault; **b** UBM images of one patient with the vault barely disappeared, while the ICL was at normal position; **c** UBM images of one patient with excessive vault, while the ICL was at normal position. The arrowes showed the vault; the red circles showed  haptics position

In this study, ICL implantation in the posterior chamber was observed at various positions and 74.1% eyes had ICL or ICL haptics dislocation such as in the ciliary process; on the top of ciliary sulcus; under ciliary sulcus; the haptics inserted in the ciliary body; one haptics under the ciliary sulcus; One haptics on the ciliary process, another haptics inserted the ciliary body; one haptics on the ciliary process, another haptics under the ciliary body; one haptics under the ciliary sulcus, another haptics inserted in the ciliary body; ICL decentralization through the UBM images of the subjects having ICL/TICL implantation. Even though, the mean vault of each kind of position observed in this study was in ideal range (250 μm -750 μm) except in three eyes with haptics on the top of ciliary sulcus (850.00 ± 70.71 μm). Among the three eyes, one eye appeared with haptics pushing forward the iris. More than half eyes (64.9%) in 134 eyes acquired ideal vault. Five eyes were with iridociliary cysts preoperatively and three eyes (60%) had ideal vault. Information regarding the population of the position of phakic IOL and the vault was seen in Table 1. Figure 3 shows the different vault when ICL had an ideal position -optical zone in the center of the pupil and the haptics located in the ciliary sulcus.

**Table 1:** ICL/TICL and haptics posterior chamber position in 72 patients (134 eyes)

Position	Eyes(%)	Vault(μm, mean ± SD)	Ideal vault eyes (250 μm–750 μm)	Insufficient vault eyes(<250 μm)	Excessive vault eyes(>750 μm)
In ciliary sulcus	29(21.6%)	573.10 ± 253.94	23(79.3%)	4(13.8%)	2(6.9%)
On the top of ciliary sulcus	3(2.2%)	850.00 ± 70.71	0	0	3(100%)
In ciliary process	17(12.7%)	453.53 ± 215.09	13(76.5%)	3(17.6%)	1(5.9%)
Under ciliary sulcus	14(10.4%)	498.57 ± 200.92	11(78.6%)	2(14.3%)	1(7.1%)
Inserted in the ciliary body	43(32.1%)	520.73 ± 329.40	22(51.2%)	13(30.2%)	8(18.6%)
One haptics under the ciliary sulcus	6(4.5%)	725.00 ± 249.78	5(83.3%)	0	1(16.7%)
One haptics on the ciliary process, another haptics inserted the ciliary body	10(7.5%)	300.00 ± 208.91	4(40%)	6(60%)	0
One haptics on the ciliary process, another haptics under the ciliary body	4(3.0%)	533.33 ± 190.35	4(100%)	0	0
One haptics under the ciliary sulcus, another haptics inserted the ciliary body	2(1.5%)	375.00 ± 388.91	1(50%)	1(50%)	0
With iris ciliary body cysts	5(3.7%)	656.00 ± 283.69	3(60%)	0	2(40%)
ICL decentralization	1(0.7%)	550	1(100%)	0	0
Total	134		87(64.9%)	29(21.6%)	18(13.4%)

**Fig. 4:**
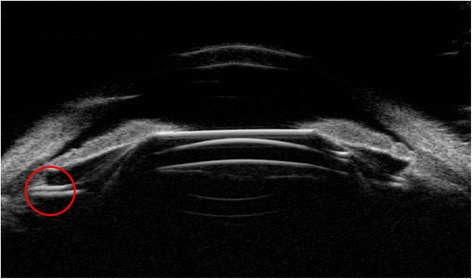
UBM images of one patient with an ideal vault, while one haptic was inserted into the ciliary body. The red circles showed the haptics position

**Fig. 5:**
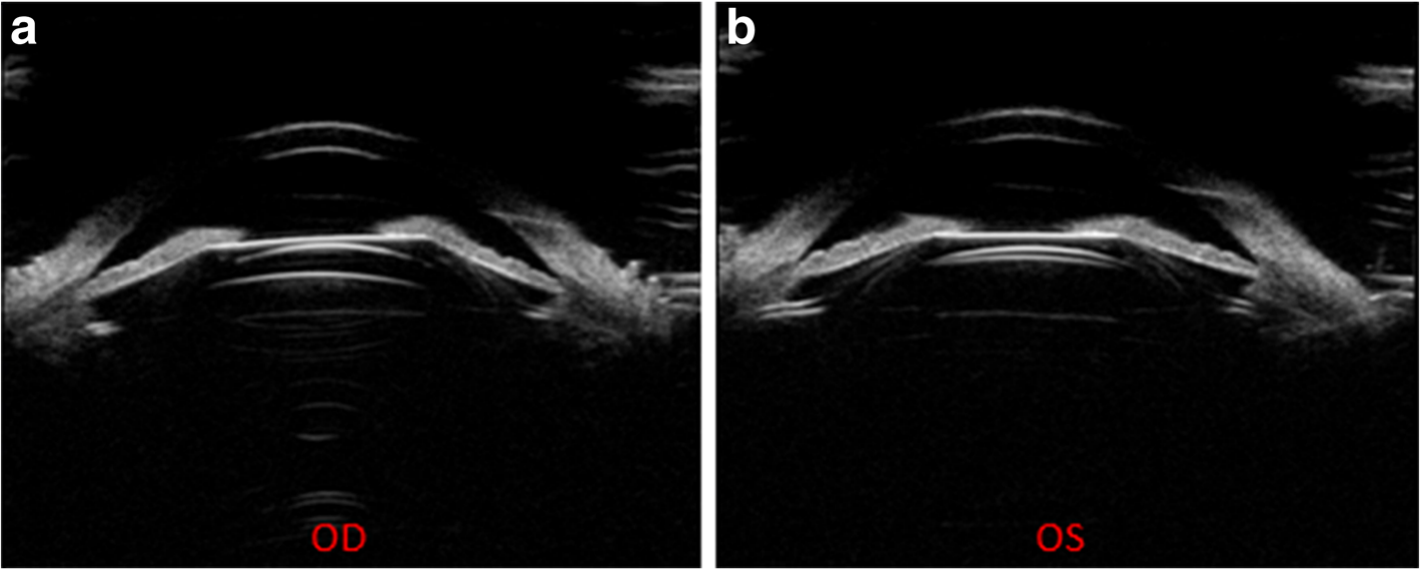
UBM images of one patient (2 eyes) with different vaults –the right eye obtained an ideal vault (**a**), while the left vault barely disappeared when the one haptic was inserted into the ciliary body (**b**)

**Fig. 6:**
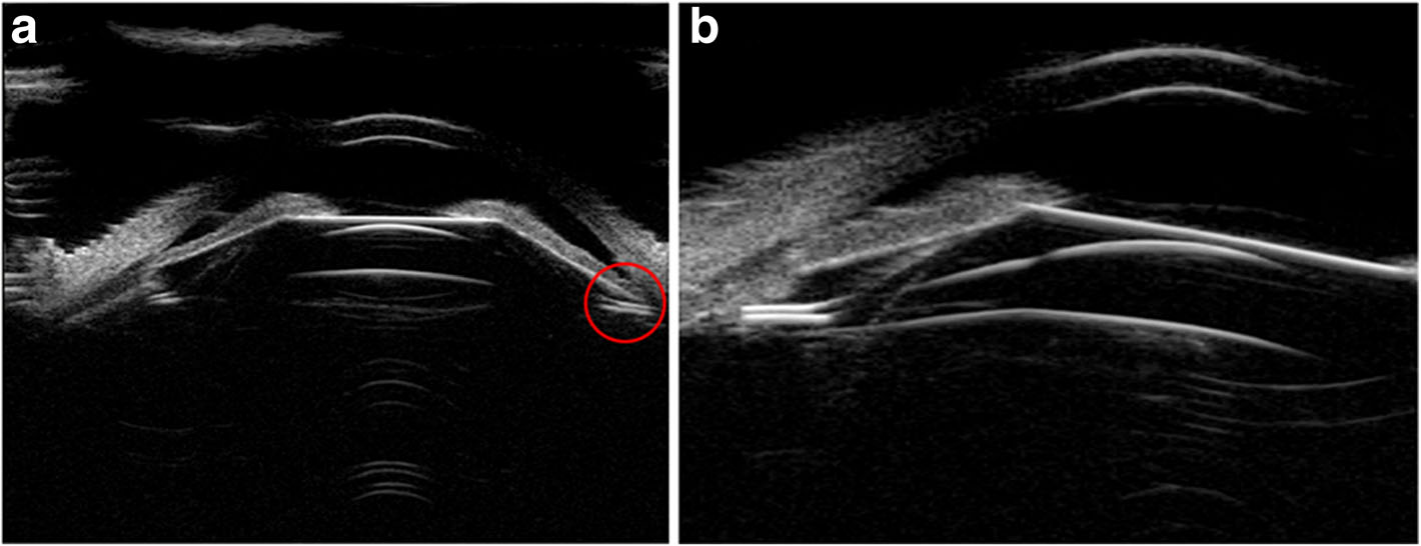
UBM images of one patient with an excessive vault, while one haptic was inserted into the ciliary body. (**a**) Full-scale images of the ICL in the posterior chamber position; (**b**) the haptics position on the nasal. The red circles showed the haptics position

Figures 4, 5, 6 shows the different vault when ICL haptics inserted in the ciliary body.

**Fig. 7:**
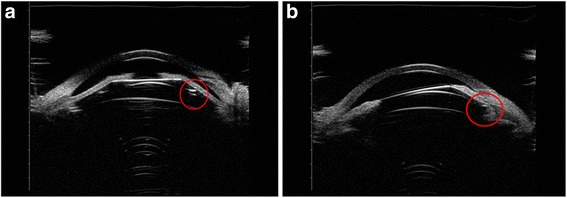
UBM images of ICL, one side haptic on the top of the ciliary sulcus and iris losing the normal arc shape because of being pushed by haptics. The vault exhibited more than 750 μm in (**a**) and (**b**). The red circles showed the haptics position

**Fig. 8:**
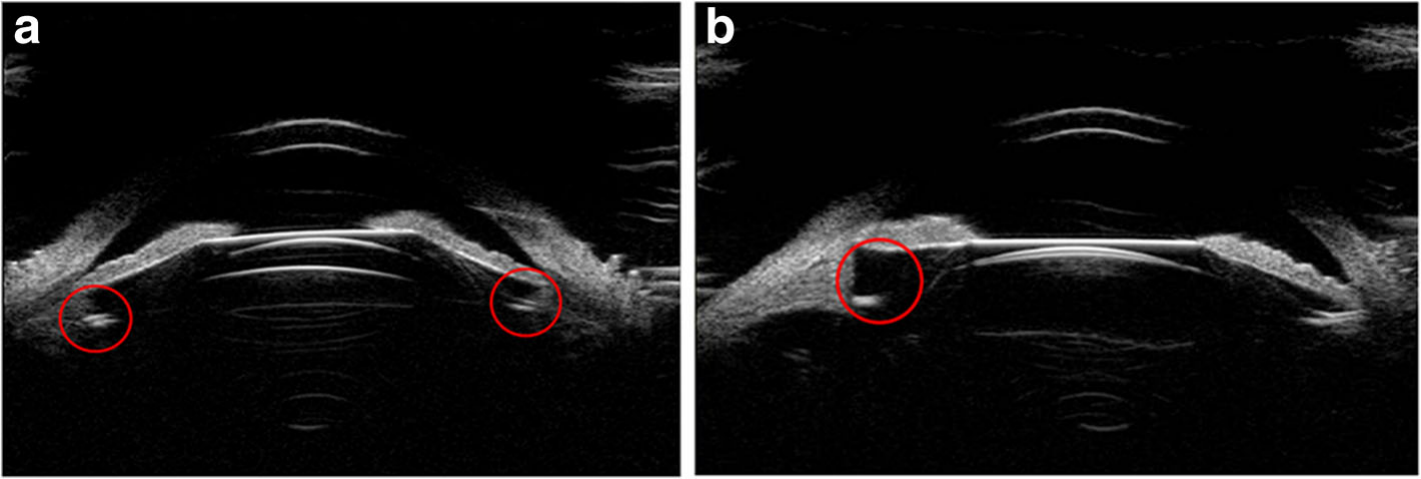
UBM images of ICL, one side haptic in the ciliary process with an ideal vault (**a**) and ciliary sulcus distortion with the vault disappearing because of the process being pushed by haptics (**b**). The red circles showed the haptics position

**Fig. 9:**
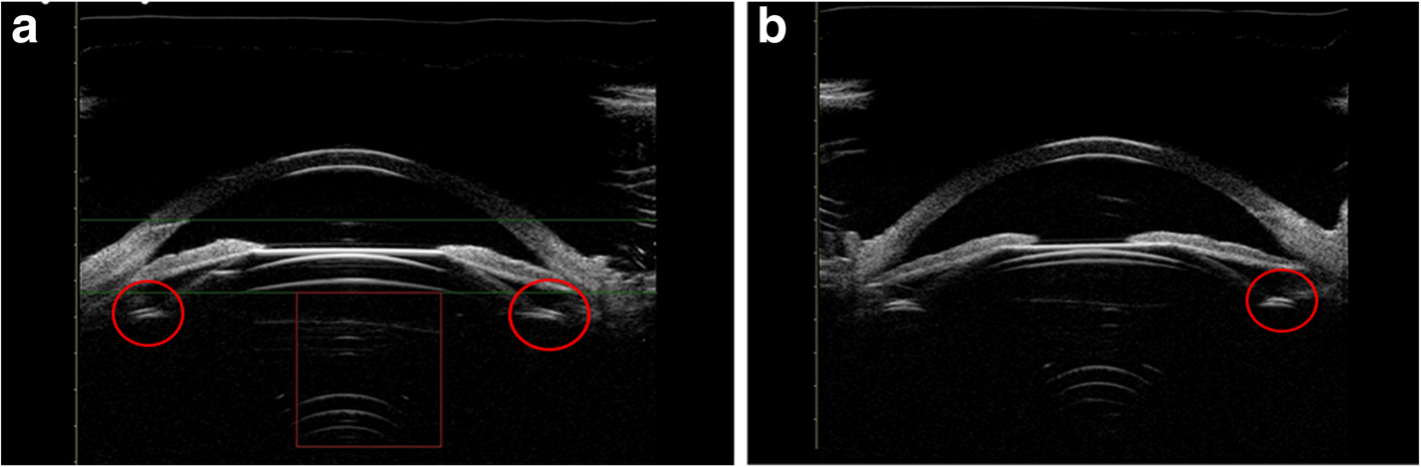
UBM images of ICL haptics under the ciliary sulcus. (**a**) bilateral haptics under the ciliary sulcus with an ideal vault. (**b**) one side of the haptics under the ciliary sulcus with insufficient vault. The  red circles showed the haptics position

Figures 7, 8, 9 shows the different vault when ICL haptics dislocation including on the top of sulcus, in the ciliary process and under the sulcus. In this study, all eyes with haptics on the top of sulcus obtained excessive vault, while another showed dislocation of haptics with no eyes exhibiting excessive vault.

**Fig. 10:**
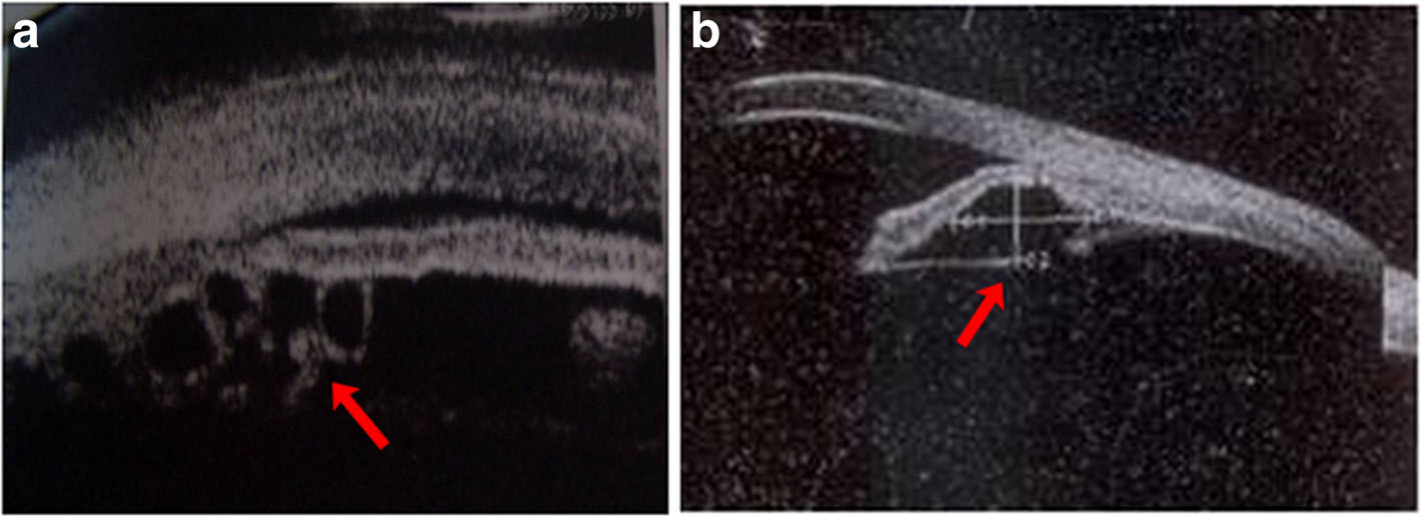
UBM images of multiple cysts (**a**) and huge cysts (diameter > 2 mm, (**b**)). Arrows showed the cysts

**Fig. 11:**
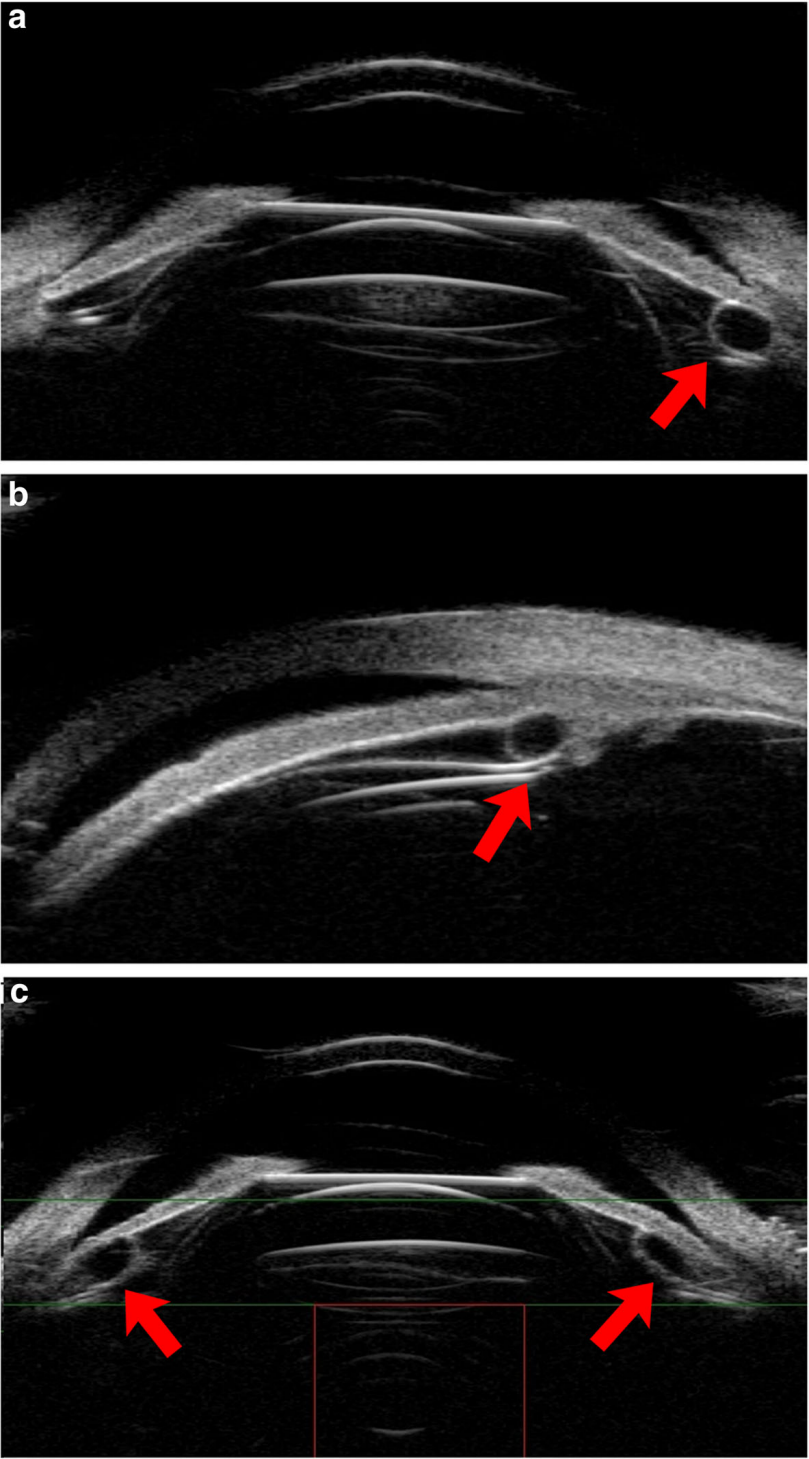
UBM images after implantation in patients with iris ciliary cysts. (**a**, **b**, **c**) listed various position of haptics. Arrows showed  the haptics

In our study, some patients underwent ICL implantation surgery with preoperative iris ciliary cysts. The incidence of iridociliary cysts ranged from 4.9% (1157 patients) to 54.3% (116 patients), which showed no consensus and the large iridociliary cysts caused anterior chamber angle closure and increased the risk of secondary glaucoma [10, 16, 17]. Most of the diameter of the cysts are less than 1 mm and have no effect on phakic IOL implantation. However, huge (diameter > 2 mm) or multiple cysts could increase the risk of ICL dislocation, the operator preoperatively should carefully estimate and avoid the cysts intra-operatively. Figure 10 showed the UBM image of preoperative iris ciliary cysts. Figure 11 showed the UBM image after implantation surgery in patients with preoperative iris ciliary cysts.

**Fig. 12:**
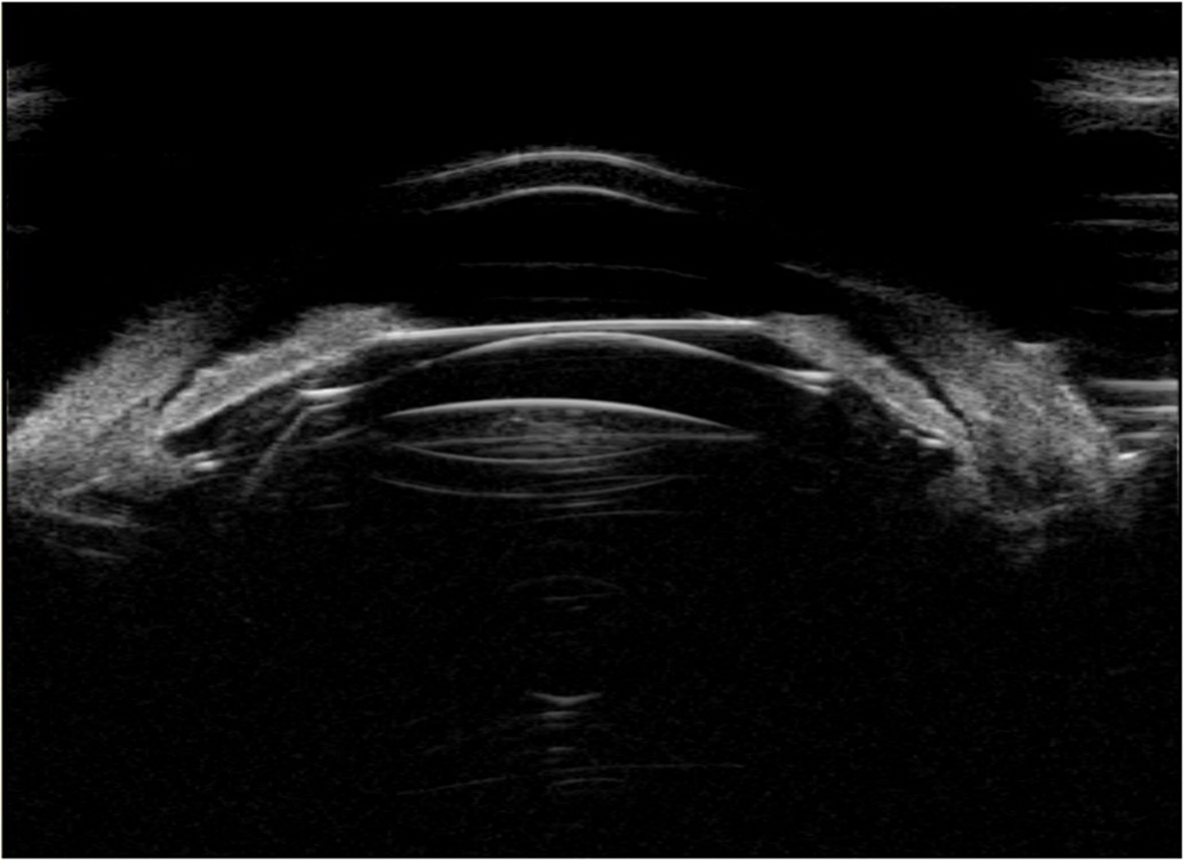
UBM images of the eye with ICL optical zone departure from the center of the pupil and an ideal vault

One case in our study demonstrated the optical zone of ICL departure from the center of the pupil and complained with severe glare. Figure 12 showed the UBM images of this patient.

## Discussion

The central vault concept is defined as the distance between the back of the surface of the ICL and the anterior crystalline lens pole. The vault is considered as an important index to evaluate the security after ICL implantation. Although most of the postoperative patients can obtain ideal vault, excessive or insufficient vaults cannot be avoided. The vault is a dynamic range but a fixed value and is influenced by many factors such as ICL size, ICL posterior chamber position, ages, pupil size [18]. The inappropriate ICL size, especially too large ICL size, caused by the preoperative white to white inaccurate measurement usually leads to shallow anterior chamber, pigmentary loss, glaucoma and corneal endothelial loss because of the excessive vault. Besides the size, the thickness of ICL was increased with refractive diopter. However, the thickness of ICL ranged from 1.19 mm to 1.09 mm according to the refractive diopter, and a difference of 0.9 mm could be ignored. Lege, B. A. [19] had reported that the vault tended to decrease with age. It was possibly related with anterior lens capsule thickening with age and decreased the distance between lens anterior capsule and back of the surface of the ICL. According to a previous study on pupil size, miosis caused by natural light could decrease the vault as compared with the drug-induced miosis [20]. The ICL in the posterior chamber position was closely associated with some complications postoperatively, but it is fully observed only by full-scale ultrasound biomicroscopy after implantation surgery and hence we implanted the ICL into the posterior chamber intra-operatively without a thorough view. In another word, the implant surgery was performed in blind, thus lead to the ICL placement does not always obtain an ideal position, especially the ICL haptics. Though UBM could be used during operation to provide a straight view, the operators always performed the implantation based on the experience due to the inconvenience of UBM and helpless for the postoperative safety and efficacy. We expect a more excellent technique to be used and prevent the dislocation of ICL and haptics.

In this study, firstly, we found that the haptics position after implantation was inserted into the ciliary body (32.1%) and 21.6% eyes with haptics in the ciliary sulcus, which was probably due to the ICL size choice preoperatively and invisible intraoperatively. Secondly, though the haptics located on various positions postoperatively, almost each group vault with different positions of the haptics was in normal range. There were 64.9% eyes obtained normal vault in total, while 21.6% eyes (29/134) with insufficient vault and only 13.4% eyes (18/134) with excessive vault. Of these, all three eyes with haptics on the top of ciliary body obtained excessive vault and one eye with haptics pushing forward the iris. This meant that the vault after surgery overall tended to be normal and were less susceptible to be excessive in various positions of haptics. This could be due to the excessive vault, which always appears after implanting too large ICL, would be adjusted during postoperative in early stage and the vault tend to decrease with time after implantation of appropriate ICL size. Thirdly, to find the relationship between follow-up time period and vault, we analyzed the vault of patients after implantation surgery who were followed-up less and more than 1 year, respectively. In 107 eyes of 58 patients were followed-up in less than a year, there were 31 eyes (29.0%) with vault less than 250 μm, and in 14 patients (27 eyes) with follow-up longer than 1 year, 16 eyes (59.3%) with insufficient vault compared to the rest of the 11 eyes with normal vault. We speculate that the vault tend to decrease with postoperative time increasing, which was in accordance with previous research [21, 22]. Among 8 patients (15 eyes) who were more than 40 years old in our study, only 5 eyes (33.3%) obtained insufficient vault and was far less than the results that were reported previously [19]. It might be due to the small sample size and we need to expand the sample size for further analysis.

How to deal with the various haptics position in different vaults is also our major concern and finding a reasonable solution would be more helpful for preoperative and postoperative estimation. In this study, we obtained the following conclusions by observing various haptics positions and took different relevant treatment depending on different situations: (a) the haptics inserting into the ciliary body (32.1%) and in ciliary sulcus (21.6%) were relatively common after the implantation surgery (b) most eyes (79.3%) with haptics in ciliary sulcus obtained ideal vault, except 4 eyes with insufficient vault and 2 eyes with an excessive vault. Among them, 2 eyes with insufficient vault were speculated to have implanted with smaller ICL due to shallow anterior chamber diameter preoperatively. No treatment was provided to the eye with excessive vault, which showed no complications of IOP rising or angle closure postoperatively. So, we could followed-up the cases with haptics in ciliary sulcus (c) the cases with ICL haptics inserted into the ciliary body were inclined to have insufficient vault, which might be due to the haptics position which reduced the distance between ICL and crystalline lens. Beyond this, some patients with vault less than 250 μm might be due to the implantation of smaller ICL size. The cases in this situation could receive follow-up observation (d) the haptics dislocation included on the top of ciliary sulcus, in the ciliary process and under ciliary sulcus. One eye in this study with haptics on the top of ciliary sulcus had iris pushed by haptics and lost the normal arc shape, so that the vault appeared far more than 750 μm. It was probably because the operator could not see the back of the iris straightly and the haptics were not incorrectly placed, which induced the ICL abnormal arching. In the case of those patients with implantation of inappropriate ICL size, we should topically instill pilocarpine to contract the pupil and observe for one week. If the vault still remains excessive and have risk of angle closure after one week, the haptics or the ICL should be adjusted through the operation. If the haptics in the ciliary process lead to the abnormal structure of ciliary sulcus, the ICL might shift downwards and the vault might usually be less than 250 μm. The haptics under the ciliary sulcus had less impact on the distance between ICL and crystalline and need follow-up observation. (e) Compared with the incidence of iridociliary cysts in the previous report (4.9–54.3%), the incidence in our study was significantly lower (3.7%). This probably might be due to small sample size. The iridociliary cysts probably effected the haptics position and induced some patients obtaining abnormal vault. For these cases, special treatment was unnecessary if the IOP was not increased. (f) One patient with the ICL optical zone decentralization in our study complained of having sever glare and the UBM images showed the vault to be in ideal range. We speculated that the postoperative visual quality was closely related with ICL position. Further research on larger sample size is warranted to conclude the results of glare or poor visual quality and explore the inner relationship between visual quality and ICL or ICL haptics position.

In this cross-sectional study, we recruited the patients who were performed with ICL V4 implantation surgery and the postoperative period ranged from 1 week to 7 years. We obtained the UBM images of every patient and mainly investigated the relationship between various haptic positions and the vault in different postoperative period. Though the changes of the haptics and ICL positions in different follow-up period of one patient were not observed in our study, the comprehensive analysis and numerous of UBM images were considered to being helpful for surgeons to deal with various ICL positions and vault. In the future, a prospective study should be performed to investigate the changes of haptics and ICL positions as the postoperative time prolonging.

## Conclusion

In conclusion, full-scale ultrasound biomicroscopy provides a straight view regarding various positions of ICL or haptics in posterior chamber after implantation. And in this study, we noticed that the location of haptics placed inappropriately (not in ciliary sulcus) might be one of the factors influencing the vault through observing positions of ICL and haptics in the posterior chamber after implantation by recording UBM images and the incidences of different vault (less than 250 μm; 250~ 750 μm; and more than 750 μm) in various situations. Though we had not abstract the exact relationship between the haptics position and vault due to the small sample size in this study, our research on ICL or ICL haptics position in posterior chamber was undoubtedly helpful for the surgeons to directly estimate the appropriateness of ICL size, the ICL implanted position and find out the possible reasons of less-than-ideal vault. Follow-up observation is usually recommended in patients with insufficient vault; but if the positions of ICL or haptics resulted in excessive vault, topical pilocarpine is applied to contract the pupil followed by observation for one week and if the excessive vault is due to a large ICL size, then the best course of action is to exchange the ICL. The accurate assessment of these cases is undoubtedly beneficial for the surgeons to accumulate more experience on evaluating the safety of the surgical outcome and dealing with the dislocation and less-than-ideal vault caused by various reasons.

## Abbreviations

ACD: Anterior chamber depth
BCVA: Best corrected visual acuity
ICL: Implantable Collamer Lens
LASIK: Laser in situ keratomileusis
PIOL: Phakic intraocular lens
TICL: Toric implantable collamer lens
UCVA: Uncorrected visual acuity
